# Medical Items and News of Interest to the Physician

**Published:** 1869-07

**Authors:** 


### Medical Items and News.

### Of Interest to the Physician.

**Note :—**

These formulæ are intended *only* for the regular practitioner of medicine, and should be employed only by him, or under his immediate supervision.

**Venerial Ulcers.**

M. Lallier recommends Iodoform for a local application to venerial ulcers.

**Typhoid Fever.**

The physicians of Liverpool use sulphurous acid, in doses of from one to three drachms, in typhoid fever, with good results.

**A New Medical College.**

Philadelphia is to have a new medical college, to be *run* by a new set of men, and not in the interest of any *clique.*

**Jefferson Medical College.**

It is reported that Prof. Pancoast of the above College has resigned the chair of Anatomy, and that D. Hayes Agnew, M. D., is to be his successor.

**Cure for Indurated Hæmorrhoids.**

The *La Union Medicale* says M. Hillairet employs suppositories containing one-tenth part of iodoform. In a few days the hæmorrhoids soften and wither.

**Threatened Abortion.**

Tincture of gelsiminium, in five drops doses every half hour, is an excellent prescription in uterine hemorrhage, being of special value in cases of threatened abortion.

**Poisoning by Mushrooms.**

M. Poulet. in a communication to the Académie des Sciences, affirms that alcohol in large doses is a veritable antidote to the poisonous mushrooms of the genus *amanite.*

**India Rubber Sponge.**

An artificial sponge, made by filling india-rubber, in a fluid state, with bubbles of gas, and then allowing it to harden, has just been introduced. It is capable of being made into pads for fracture and hernia, and is very elastic.

**Iodide of Sodium in Lead Poisoning.**

The iodide of sodium is advised by M. Rabuteau in treating lead poisoning, instead of iodide of potassium. The former is as active an eliminant as the latter, and no ill effects are produced.

**Diarrhœa in Infants.**

Dr. Smith, in the *Med. News and Lib.*, recommends the following prescription, if the bowels are rather loose, with dark, slimy, offensive stools: R. Tinct. Opii ♏viij; Ol. Ricini, ʒj; Syrupi Zingib., Mucilage of Acaciae āā℥j—M. Sig:—One teaspoonful three times daily.

**Senile Catarrh.**

Dr. J. Curran Waring (*The Practitioner.*) has found cannabis an invaluable remedy in catarrhus senilis. He administers it in grain doses, gradually increased. He believes that as an anodyne it is immensley superior to every other drug.

**Blennorrhoea and Leucorrhoea.**

Dr. Bogs of the Indian Service, recommends the following formula as an injection:—

Alcoholic Tinct. of Iodine 45 drops; pure liquid carbolic acid, six drops; glycerine, one ounce; distilled water, five ounces. In blennorrhœa and leucorrhœa, this mixture is said to be superior to tar-water.—*Record.*

**New Anæsthetic.**

The *La France Medicale* says that the Protoxide of Azote is being employed as an anæsthetic by the physicians of Paris, with great success. It produces complete insensibility in two minutes, and is respired without difficulty or repulsion.

**Prurigo.**

The following ointment is recommended by Dr. Charvet, through the *Bull. Len. de Therap.* viz:—

Axrange, simple or camphorated, sixty parts; citrine ointment three parts; mix. A small portion to be spread over the affected surface.

**Great Loss.**

Dr. Pope of the St. Louis Medical College lost his very extensive collection of specimens in Natural Science, by fire, recently.

He had the largest anatomical collection too, in this country. We spent many profitable hours in his cabinet when a student, and are sorry to learn of its destruction.

**New Dressing for Wounds.**

In Belgium they dress wounds by applying a piece of sheet-lead—one fifth of an inch in thickness—to the seat of injury, causing it to assume the proper shape by pressure. This dressing is kept in place by means of adhesive plaster, and a current of cold water is passed over the surface of the flesh twice a day.

**Malarious Fever.**

The surgeons in Mauritius speak highly of carbolic acid in intermittent fever. One grain of the pure medicinal acid in one ounce of water, with a little brandy or bitter infusion, was administered as a medicine three times per day, and with, it is asserted, remarkably good effects, the relapses being less frequent than after the use of quinine.

**Whooping Cough.**

An experienced practitioner, in an article published in the *Canada Medical Journal*, says that the following prescription is the best he has ever used: R. Ammon-bromid. ʒj.; acid hydrocyan. dil., ♏xx; tinct. sem. stramonii, ♏xx; water and syrup ℥ jv. M. A teaspoonful three times daily to a child two years old, will often relieve in twenty-four hours.

**Treatment for Nervous Headache.**

The following remedies in the treatment of this affection are recommended by Prof. W. A. Hammond:—Oxide of zinc; ordinary dose, two grains three times a day, after meals; maximum dose five grains. It is best given in form of pills. The Sub-carbonate of Bismuth will sometimes take the place of oxide of zinc. Dose, two grains after each meal.—*Record.*

**Pruritus Vulvæ.**

It is stated (*Gazette des Hopitaux,*) that this annoying affection is often entirely cured and always diminished by a lotion consisting of five parts of corrosive sublimate dissolved in fifty parts of alcohol. A teaspoonful of this solution is to be diluted with a pint of tepid water, and applied as a wash to the parts several times in the day.

**Catarrh of the Bladder.**

This disagreeable chronic complaint is often very obstinate; it may therefore just be stated that M. Malloz has found the following solution, injected into the bladder very efficatious: R Aqua Dist. ℥ x: Tinct. Iodine, gtt. xiv; Pot. Iod. gr. xv. When the pain is very annoying, add fifteen grains of extract of belladonna to the above. He has employed carbolic acid, nitrate of silver, and hyposulphate of soda, with advantage.

**Treatment of Cataract by Phosphorus.**

M. Tavignot, a well known and much esteemed Parisian Oculist, has lately published in the *France Medicale*, a series of cases which would go far to prove that frictions on the forehead with phosphorated oil, and instillations of the same into the eye, may contribute to the melting away of the hardened lens or capsule, and the restoration of sight, without the usual operation. These are worthy of attention and should be read by all those who take an interest in ophthalmology.

---

**Syphilis Treated Hypodermically.**

Dr. Hamer's (*Humbolt Med. Archives*,) reports further satisfactory results from the hypodermic injection of one-eighth grain doses of the bichloride of mercury dissolved in water, in constitutional syphilis. Under the use of this remedy once daily, the worst symptoms rapidly disappeared. In this treatment there is said to be less danger of unpleasant local effect,—inflammation, suppuration, and abscess—if the injections are made through the thicker skin over the loins.

---

**Treatment of Diarrhœa.**

℞.
Salicin gr. v.
For one powder.

To be taken every four or six hours.

In cases of diarrhœa with clean tongue, which will not yield to opiates, astringants, or stimulants, either singly or combined, and which probably depend on a want of tone in the intestine. In these cases the receipe has often stopped a diarrhœa that appeared fast hurrying the patient to his grave.—Therapeutical Bulletin—Phila., *Med. and Surg. Reporter.*

---

**Sciatica Treated by Acupuncture.**

The *Boston Med. and Surg. Journal*, records an interesting case of sciatica in which acupuncture was successfully tried. The patient had tried all the usual remedies for unilateral sciatica, without relief. Twelve fine steel needles were inserted in the direction of the nerve along the ischio-trochanteric fossa as follows: After stretching the skin tense with the left hand, the needle was seized by its head and passed about half an inch through the skin, in a boring or rotary manner. All the needles were successively applied and left in place half an hour, when they were extracted. The patient then was requested to get up and walk, and to his astonishment, he did so with ease and without pain. The cure was radical.

**Bronchitis and Phthisis.**

Dr. J. M. Da Costa (*Med. and Surg. Reporter*) recommends the following formula as a sedative mixture for the cough of phthisis:

℞.

Morphiæ acetatis, gr. ij.

Potassii cyanidi, gr. j.

Acidi acetici, f ʒ j.

Ext. pruni Virginianæ fl.

Misturæ acaciæ āā f ℥ ij.

M.

Sig:—One teaspoonful six times pr. day.

---

**Purulent Ophthalmia.**

In new born infants use the following collyria:

℞.

Hyd. chl. corros. gr. j.

Ammoniæ Muriatis gr. jv.

Aquæ Rosæ f. ℥ vj.

Fiat Sol.

Wash the eyes several times during the day with this solution.

---

**Ulcers, Eczema, &c.**

The following ointment will be found very beneficial:

℞.

Zinci oxidi, ʒ jss.

Amyli, ʒ j.

Cerati adipis,

Glycerinæ, āā ℥ ss.

M. Prof. Pancoast.

---

**Chronic Glandular Tumors.**

Dr. J. M. Da Costa prescribes the following

℞.
Cadmii iodidi, gr. x—xx.
Unguent. bell. ℥ vj.
Glycerine, f. ℥ ij.
*Med. and Surg. Reporter.*

---

**Diabetes.**

Dr. Da Costa is in the habit of prescribing the following formula in diabetes, with great success;

℞.

Acidi Carbolic flud. ♏ xxiv.

Glycerinæ,

Aquæ, āā f. ℥ jss,

♏.

Sig:—Teaspoonful ter die.

---

**Gleet Treated with Carbloic Acid.**

Dr. Whitehill, (*Humbolt Med. Arch.*) stated to the St. Louis Medical Society, that he had, about a week before, been consulted for a case of gleet, of over two years duration, that had resisted all previous treatment.

He ordered, internally tr. ferri chlor. gtt. x. and tr. canthar. gtt v., three times daily, in sweetened water, and the injection morning and evening, with a long nozzled syringe, of a-half ounce of a mixture or carbolic acid, ʒ. j. aq. ext. opii., ʒ ij., and glycerine, ℥ iij., to infus. matico, ℥ v.; the injection to be used after fully evacuating the bladder and to be retained at least ten minutes, and then micturation to be avoided as long as possible.

The first morning after injection the discharge was very materially increased; the second morning it was still increased in quantity, but no longer purulent; the third morning there was no discharge perceptible; on the fourth there was "about a-half drop, that looked like the white of an egg", and since then there has been none. The injection produces no pain.

---

**Treatment of Sunstroke.**

Dr. MacLeon, having had an extensive experience in the treatment of this, in India, recommends the following: The person stricken down by heat, is to be at once removed to a shade, stripped and douched with cold water over head, neck, and chest. By this means a powerful impression is made upon the cutaneous nerves, the effect of which is to set suspended respiration in motion first by catches and gasps, finally in a regular manner. If the heat of the skin be high, the cold douches should be repeated. The patient should also drink freely of ice-water. Amonia should also be inhaled, from time to time. As soon as sensibility is restored it is well to give a purgative. If sensibility is not restored by the above means, the head should be shaved and a blister applied, and in the convulsive form, chloroform may be inhaled.—*Lancet.*

---

**Popliteal Aneurism.**

The *Western Jour. of Med.* records a case of popliteal aneurism cured by manipulation, in the following manner:—"A patient, æt. 25, was injured in the leg by a fall sustained in rolling logs. To the fall and the twist of the leg he attributed the swelling and pain about the knee joint. The swelling increased until it became of the size of a large orange. The aneurismal bruit was very distinct, and all the symptoms left no doubt as to the character of the tumor. In the treatment the leg was flexed strongly upon the thigh, and firm digital compression was kept up upon the femoral just below Pouparts ligament. At the end of thirty minutes, only a slight thrill could be detected. The compression was continued for sixty-eight minutes, when the leg was secured to the thigh by a strong band of adhesive plaster. It was supposed that the compression already used was sufficient for the formation of the clot, but to make it certain the flexion treatment was continued for some time longer. During the succeeding night the patient had no sleep in consequence of the severe pain experienced. The temperature of the foot and leg was sensibly diminished and the sensibility of the parts greatly increased. On the next day after the operation the adhesive plaster was removed and the leg extended, which afforded great relief.

On the second day the tumor was sensibly diminished in size, and on the third no pulsation could be felt. From this time the patient improved, and was discharged on the ninth day after the operation, cured.

---

**New Treatment for Rheumatism.**

At St. Mary's we have noted of late several patients recovering from acute rheumatism, and learned something of the plan which has been adopted by Dr. Sibson during the last year in all cases, without exception. It may be described as embracing three points. 1. Removal of pressure and tenison of joints. 2. An even and warm temperature. 3. Removal or relief from pain. To accomplish the first of these ends, the patient keeps his bed, and his joints are puffed in cotton wool and flannel, a cradle being placed where the weight of the bed clothes is painful. For the second, the patient wears a flannel dressing-gown, and the blankets touch the skin of the lower extremities, sheets being placed only over the upper part of the head. For the third, the linimentum belladonnæ [R. Ext Belladonnæ ʒ. j. Linimentum Saponis, ʒ. viij.] is applied to painful joints, and covered over with wadding. Occasionally, when the pain is very excessive, from an eighth to a quarter of a grain of morphia is injected subcutaneously. For the rest, he has now and then found it useful to apply a leech or two to a swollen joint or to the cardiac region. In cases where there appears to be a gouty complication, Dr. Sibson employed a little iodide of potassium; but apart from this he does not give any potash to his patients. He tells us, in answer to an inquiry, that he finds the urine rarely containing acid after the first few days of treatment. As regards food, his experience and practice are not a little interesting. The patient is allowed from the first, roast meat, rice pudding, and porter. We ascertained moreover, from inquiry of the nurses, that this diet was not only ordered by the Doctor, but was consumed by the patient, with very rare exceptions. Some patients to whom we spoke confirmed this statement; and added, also, strong testimony to the immense relief derived from the application of belladonna in the way described. The nurses said that the patient generally slept well at night.—*Lancet.*

### A Case of Petrifaction.

The following singular case of petrifaction was recently published in the *Criminal Zeitung*: Amos Broughton, of Wayne county, Iowa, died six years ago, and recently on disinterring the body it was found in a state of petrification, like a marble statue. Every feature was perfect, and the whole face life-like. The weight of the statue was 400 pounds. A similar case occurred in Woodlawn cemetery, in this city about a year ago. The subject was a little child, disinterred after a year's burial, and was found converted into solid stone.

BAD WINE AND WHISKEY.—The London *Lancet* says: The impunity with which our ancestors used to drink so much port and claret is less a proof of the strength of their constitutions than of the excellence of the liquor imbibed—not even the strongest of them being one whit better able than ourselves to indulge with any freedom in the so called "port" of second rate hotels, or the "sherry" that scares visitors in middle-class houses on its entrance with the luncheon-tray. Ligneous port and igneous sherry, however, are innocuous compared with the vitriolic whisky, or the fiery gin with which the lower orders are accustomed to refresh themselves; and no movement deserves more encouragement from medical practitioners, or, for that matter, from the payer of poor-rates, than that which places good, wholesome wines within their reach.

—Every railroad train in Sweden is provided with an efficient medical staff and complete pharmacy, so that in case of accident, no time is lost in ministering to the wants of the wounded.—*Exchange.*

Must have dangerous roads in Sweden—wonder whether they have discharged all their old Conductors, *a la* Erie ?

### Trbutes to Vanity.

WHAT OUR NEIGHBORS SAY OF US.

A SHARP INSTRUMENT.—The BISTOURY—a quarterly medical journal, edited by Thad S. Up De Graff, M. D., of Elmira. N. Y. The best edited medical journal in this country—send for a copy and see.—*Indianapolis (Ind.) Journal.*

THE BISTOURY.—We have read, with considerable pleasure, a journal named as above, and edited by Thad. S. Up De Graff, M. D., at Elmira, N. Y. In this age of humbuggery, just such a magazine as the BISTOURY is needed to keep folk's eyes open. A more spicy and readable journal does not come to our table.—*St. Louis (Mo.) Republican.*

THE BISTOURY, one of the most entertaining and instructive medical and miscellaneous publications of the day, will make its appearance on the first of April in a new and improved form. Thad. S. Up De Graff, M. D., of Elmira, is its editor, to whom all orders for it should be addressed.—*Corning Democrat.*

DR. UP DE GRAFF'S Bistoury comes to us with 32 pages of reading matter and a new cover, and is a really readable work. Thad. is a genius, and we should not be surprised to see the BISTOURY taking rank before many years with some of the old established medical magazines. At 50 cents a year it is cheap and valuable reading matter.—*Gazette, Troy, Pa.*

THE BISTOURY.—We received, this week, No 1 of the fifth volume of this most excellent Medical Journal, published quarterly, by Dr. Up De Graff, at Elmira, N. Y. The price of subscription for this little work is 50 cents, and worth double the sum, to every practicing Physician.—*Brownsville (Tenn.) Bee.*

DR. THAD. S. UP DE GRAFF'S BISTOURY for April made its appearance in a neat pamphlet form of 32 pages. He is now circulating many thousands copies of his publication. We like the way Thad talks in this book of his, and trust he may live to "spout" many years yet. Being a true and courteous Sir Knight, of course he is sensible, and being sensible he makes his book readable, and useful in matter of information. Chalk us down for a "regular," Doc.—*Northern Pennsylvanian, Great Bend, Pa.*

THE BISTOURY.—No. 1 of the 5th Volume of this excellent Quarterly has reached us. We have no hesitation in saying that so much real practical sense and useful information to all as may be found within the covers of this Magazine of 32 pages, is seldom found in books of thrice its size. There are articles on "Prodigy Making," "Springs," Thos. K. Beecher, &c., &c. Six letters with appended answers, a fine lot of clipping; and a few pages of editorials in the editor's spicy vein. Dr. Up De Graff editor and proprietor, Elmira, N. Y. Price 50 cents a year.—*Wellsboro Agitator.*

---

THE BISTOURY.—We have received the April number of Dr. T. S. Up DeGraff's Magazine—a neat pamphlet of thirty pages, changed from quarto form. Its contents are of a varied and interesting character, consisting of editorials, correspondence, clippings, &c. It has a number of contributors well known to our citizens, which will add materially to the interest of the book. A number of our business men are represented in different parts of the work, and altogether it makes a valuable and entertaining magazine for the people. It is afforded at the very cheap price of fifty cents a year, which will enable every one to procure a copy. We wish the Doctor success with his new publication.—*Saturday Evening Review.*

---

DR. T. S. UPDEGRAFF, of Elmira, has changed the form of his quarterly publication called the BISTOURY. It is now a neat pamphlet of about thirty pages, with a cover, and is filled as usual with interesting original and selected articles. Dr. Up DeGraff has become noted as an operating surgeon, and in the treatment of eye and ear diseases; and the BISTOURY is his organ of communicating with the public. Much of the matter is of general interest. Terms of the BISTOURY half a dollar per year. He offers remarkable inducements for Agents to raise Clubs, so as to raise it from 20,000, the present circulation, to 100,000.—*Corning Journal.*

---

THE BISTOURY.—Number One of Volume Five of the BISTOURY is before us, and with the new volume commences a new series. Doctor Up DeGraff not only possesses eminent abilities in his profession, but as an editor he has few equals in getting up an interesting, entertaining and instructive publication. In addition to the original and selected articles by its editor, the BISTOURY will contain matter from the pens of the following contributors: Rev. T. K. Beecher, W. H. Gregg, M. D., Robert Hall, Esq., Prof. J. D. Steele, B. H. Pratt, A. M., and Dr. P. Hays.

The BISTOURY will be devoted to the enlightenment of the family circle upon medical subjects, and the exposition of charlatanism and humbuggery wherever found, and in addition to the medical items and news, which will be culled from the standard medical journals of the world by the editor, it will always contain articles upon health, reform, education, chemistry, medicine and general science, from some of the gentlemen mentioned as contributors.

The new series of the BISTOURY is a journal of thirty-two pages, and is published quarterly, on the first of April July, October and January, at fifty cents a year. It is edited by that genial gentleman, Thad. S. Up DeGraff, M. D, and we wish both him and his publication an abundant measure of success.—*Elmira Daily Advertiser.*

---

THE BISTOURY IN A NEW FORM.—This familiar publication for April, 1869, makes its appearance in a new form. It is now a double-column magazine of thirty large pages. It has a most attractive cover, and its inside contents are neatly made up, consisting of communications from the following well-known and talented home writers: B. H. Pratt, A. M, Thos. K. Beecher, Prof. J. D. Steele, Robert A. Hall; Correspondence, Clippings and Editorials, by the editor, Thad. S. Up De Graff, M. D. The subject matter of the magazine is of a most useful and interesting character, while the communications give to the journal a distinctive literary character. The BISTOURY in its present shape is certainly greatly improved in typographical neatness, and in the order and quality and variety of its contents. The BISTOURY is now five years old. It was originally started as a free medical journal—less than three thousand being published in the commencement. In the meantime it has been repeatedly enlarged and improved, attaining a circulation of 20,000 copies. With the change of form there has also been a change in the subscription rules. Heretofore the journal has been free. Now it is offered at the low rate of fifty cents a year. The publisher and editor announces large cash premiums for subscribers by means of which his subscription list, in a great measure, will no doubt be kept up, and the character of his journal improved. It is certainly a publication of merit and value, deserving of a place in every family.—*Elmira Daily Gazette.*

